# Mindfulness as a Buffer of Leaders’ Self-Rated Behavioral Responses to Emotional Exhaustion: A Dual Process Model of Self-Regulation

**DOI:** 10.3389/fpsyg.2018.02498

**Published:** 2018-12-07

**Authors:** Megan M. Walsh, Kara A. Arnold

**Affiliations:** ^1^Edwards School of Business, University of Saskatchewan, Saskatoon, SK, Canada; ^2^Faculty of Business Administration, Memorial University of Newfoundland, St. John’s, NL, Canada

**Keywords:** mindfulness, transformational leadership, abusive supervision, dual process theory, perspective taking, negative affect

## Abstract

In this study, we investigate the self-regulatory role of mindfulness in buffering the relationship between leaders’ emotional exhaustion and self-rated leadership behavior (transformational leadership and abusive supervision). Specifically, we propose that leader mindfulness buffers the relationship between emotional exhaustion and both negative affect and impaired perspective taking, which link leader emotional exhaustion and leadership behavior (i.e., moderated mediation). Using a time-lagged survey of leaders (*N* = 505) we found that leader perspective taking and negative affect mediated the relationships between emotional exhaustion and self-reported leadership behavior. Furthermore, we found that leader mindfulness buffers the relationship between emotional exhaustion and negative affect, which weakened the mediated relationship between emotional exhaustion and both transformational leadership and abusive supervision. However, leader mindfulness did not moderate the relationship between emotional exhaustion and perspective taking. Theoretical and practical implications are discussed.

## Introduction

Mindfulness, defined as “a receptive attention to and awareness of present events and experience” (Brown et al., 2007, p. 212) has been part of Buddhist philosophy for centuries. In addition to its spiritual roots, mindfulness is quickly gaining attention in psychology and management as a potential tool for improving well-being and performance at work (Good et al., 2016). One area of particular importance has been research demonstrating that mindfulness can interact with challenges at work to predict leader behavior (e.g., Liang et al., 2016). We draw upon Glomb et al. (2011) mindful self-regulation framework, in addition to dual process models of self-regulation (Strack and Deutsch, 2004), to further investigate the moderating role of leader mindfulness. Specifically, we suggest that mindfulness enables leaders to regulate cognitive and affective responses to emotional exhaustion, which in turn is associated with positive leader behavior.

Leader behavior is of critical importance for workplaces, as leader behavior has direct and indirect relationships with employee well-being (e.g., Arnold, 2017) and organizational functioning (Judge and Piccolo, 2004). A key antecedent to leader behavior has been leader resource depletion, which has been conceptualized in varying ways such as general stress, strain (an outcome of stress where demands are excessive; Scott and Charteris, 2003) and chronic indications of strain such as depression (e.g., Burton et al., 2012; Byrne et al., 2014; Zhang and Bednall, 2016). We chose to investigate emotional exhaustion, defined as “feelings of being emotionally overextended and exhausted by one’s work” (Maslach et al., 1996, p. 4), as a predictor of leadership behavior for three reasons. First, emotional exhaustion is an indication of strain that is linked explicitly to one’s work role (e.g., being a leader) (Maslach et al., 1996). Second, the incidence of leader emotional exhaustion is quite high as evidenced by recent studies (e.g., Arnold et al., 2017); thus, better understanding the relationship between emotional exhaustion and leader behavior, and how to potentially address its negative outcomes, is critical. Third, emotional exhaustion has been shown to predict impaired cognition and affect (Lazarus, 1999; van der Linden et al., 2005), which in turn are regulatory processes that predict leader behavior (Liang et al., 2016). As we will argue throughout this paper, mindfulness can buffer the relationship between emotional exhaustion and these regulatory processes (perspective taking and negative affect) to ultimately predict a weakened relationship between emotional exhaustion leader behavior. Specifically, in this study we focus on transformational leadership and abusive supervision as self-rated behavioral outcomes.

Transformational leadership is characterized by four highly correlated dimensions: inspirational motivation, individual consideration, idealized influence, and intellectual stimulation. Inspirational motivation is the degree to which a leader motivates followers by communicating a vision. Individual consideration is characterized by recognizing followers on a personal level and giving them unique development and support. Idealized influence is the degree to which a leader acts as a charismatic role model for followers. Finally, intellectual stimulation is characterized by encouraging followers to be creative and to think outside the box (Bass and Riggio, 2006). Abusive supervision is defined as leaders engaging in “the sustained display of hostile verbal and non-verbal behaviors, excluding physical contact” (Tepper, 2000, p. 178) and is considered a universally destructive form of leader behavior (e.g., Mackey et al., 2017).

We have chosen to investigate these two types of leader behavior for two reasons. First, the small number of studies investigating emotional exhaustion (and related constructs such as depression) as predictors of leadership behavior have tended to focus on these two ‘extremes’ of leadership behavior (e.g., Burton et al., 2012; Byrne et al., 2014; Lam et al., 2017). Thus, we aim to extend previous research in this area by further investigating the regulatory factors linking emotional exhaustion and both transformational leadership and abusive supervision. Second, while some studies have focused on how mindfulness can directly predict positive leader behavior (e.g., Pinck and Sonnentag, 2018), research on self-regulatory frameworks would suggest that it is also important to further understand the moderating role of mindfulness for leadership in particular (Liang et al., 2016). Specifically, we focus on the moderating role of mindfulness in relation to emotional exhaustion and both positive leadership behavior (i.e., transformational leadership) and negative behavior (i.e., abusive supervision). Abusive supervision has not been the focus of as much mindfulness research as transformational leadership (see Liang et al., 2016; Lange et al., 2018 for notable exceptions). Thus, our study’s purpose is to demonstrate the potential for mindfulness to predict a weakened relationship between emotional exhaustion and transformational leadership/abusive supervision by moderating the relationship between emotional exhaustion and its cognitive and affective processes.

### Mindfulness and Leadership

Mindfulness is a state of consciousness that can be strengthened through practice (e.g., meditating) and is also considered a capacity that can vary naturally (i.e., dispositional or trait mindfulness; Brown and Ryan, 2003). In this research, we are investigating the benefits of trait mindfulness, which we conceptualize as a tendency toward mindful awareness that can be improved through mindful practices. Research has validated, using samples of Zen practitioners, clinical, and general populations, that trait mindfulness is a distinct form of attention and awareness that can be cultivated through practice (Brown and Ryan, 2003). Furthermore, Glomb et al. (2011) suggest that both state and trait mindfulness are able to enhance behavior at work by optimizing self-regulation, which suggests that the potential for mindfulness to buffer the relationship between stressors/strain at work and behavior is central in explaining its positive workplace outcomes.

Emerging research has suggested that mindfulness can directly and indirectly predict positive leader behavior (Reb et al., 2015). For instance, studies have found that leader mindfulness positively predicts transformational leadership behavior and negatively predicts abusive supervision (Lange et al., 2018), and that leader mindfulness predicts positive employee outcomes through mediating constructs such as employees’ psychological need satisfaction and interpersonal justice (Reb et al., 2014, 2018). Furthermore, some have found that leader mindfulness predicts positive leader behavior through leader-level mediators such as leader self-efficacy (Carleton et al., 2018). These studies have been influential in demonstrating the potential for leader mindfulness to predict positive leader behavior and, in turn, positively impact the employees who follow them (e.g., Reb et al., 2014).

However, an alternative theoretical perspective suggests that leader mindfulness may also predict leader behavior by moderating the self-regulatory processes that link workplace experiences to leader behavior. For example, one study found that mindfulness can predict lower levels of abusive supervision by interacting with leader hostility toward subordinates (Liang et al., 2016). In addition, Long and Christian (2015) found that employee mindfulness moderated the relationship between experiences of injustice and both the cognitions and emotions that followed those experiences. Ultimately, the moderation of these self-regulatory processes predicted a lower likelihood of employee retaliation for employees who were more mindful. Although not focused on leaders, Long and Christian (2015) findings in conjunction with Liang et al. (2016) investigation of leader mindfulness and abusive supervision suggest that mindfulness has an important moderating role, which ultimately predicts improved behavior for both leaders and employees.

Both of these streams of research are important and provide insights about mindful leadership. However, self-regulatory models have been, so far, the most robust when considering workplace behavior as an outcome, in addition to extensive research on the self-regulatory role of mindfulness in clinical psychology (Brown et al., 2007; Glomb et al., 2011; Good et al., 2016). Indeed, mindfulness scholars have often argued that mindfulness helps individuals to better regulate cognitions and affect *in relation* to challenges in their environments (Howell et al., 2010; Glomb et al., 2011). In other words, research and theory strongly suggest that mindfulness predicts behavior not by directly reducing negative affect and directly improving thought processes, but by buffering the negative thoughts and affect that stem from experiencing strain at work to, in turn, improve behavioral responses to that strain. Thus, we propose that the ability for mindfulness to predict positive leadership behavior primarily rests upon its regulatory role in moderating the internal responses leaders have to the strain they are already facing, rather than to eliminate or inherently change affect and cognitions themselves.

To elaborate further, Glomb et al. (2011) suggest that there are two key processes that are associated with mindfulness that help explain the potential relationship between mindfulness and individuals’ behavior at work. First, mindfulness reduces automatic responses to internal and external challenges that individuals face. When experiencing challenges in one’s environment, for example, individuals have a tendency to defer to heuristic-based thought processes. Mindfulness, however, allows ones’ awareness to remain broad and flexible in relation to adverse thoughts and experiences. Second, mindfulness allows individuals to separate themselves from their immediate experiences (i.e., decoupling), which allows them to view their own struggles or adversity in a more objective, detached manner.

The increased response flexibility and decoupling associated with mindfulness also improves the secondary regulation of thoughts and affect, which is closely related to behavior at work (Glomb et al., 2011). Self-regulation is the extent to which individuals control urges and impulses that are incompatible with societal norms (Muraven and Baumeister, 2000; Barnes et al., 2015). In relation to self-regulation, the decoupling and reduced automatic responses detailed above are core processes associated with mindfulness that in turn, help to buffer the relationship between workplace experiences and both cognition and affect. Glomb et al. (2011) outline many possible self-regulatory processes that predict behavior, and whose predictors potentially interact with mindfulness. However, we chose two of these processes that are known to drive leader behavior: Negative affect and perspective taking. We elaborate on these processes shortly. As will be discussed in the following sections, these processes not only predict how leaders behaviorally respond to emotional exhaustion, but are also weakened by a leader’s mindfulness.

### The Buffering Role of Mindfulness

In an effort to better understand the antecedents of leadership behavior, dual process self-regulatory frameworks have been useful in explaining how and why leaders enact various behaviors in response to challenges at work (Liang et al., 2016). The central tenet of dual process models is that individual behavior is determined by two separate processes that operate in parallel: impulsive and reflective. The impulsive system is automatic and operates with very little conscious awareness, although people can be aware of parts of its process (e.g., perceiving a pleasant feeling). The reflective system is a higher order system that complements the impulsive system by performing executive functions such as making evaluations and judgments. The interplay of both systems determines, in conjunction with key moderators (such as mindfulness), individual behavior (Strack and Deutsch, 2004). Although dual process models are often used to explain responses to acute events, it is also appropriate to apply dual processes to chronic events (i.e., experiencing emotional exhaustion), as research has applied this model to habituation and impression formation (Groves and Thompson, 1970; Brewer, 1988).

We chose to investigate the mediating role of negative affect and perspective taking as key components of the impulsive and reflective systems, respectively. We argue that these two components link emotional exhaustion and leader behavior. We define negative affect as a general dimension of “aversive mood states,” which are closely related to emotional reactivity (Watson et al., 1988, p. 1063). In terms of negative affect, Glomb et al. (2011) outline that there are various mindfulness-based processes that relate to work-related behavior in general. Amongst these are affective processes, which are closely related to leadership (e.g., Arnold et al., 2015). There is a growing recognition that leadership is emotionally complex and that both emotions and affect are closely related to leader behavior (e.g., Humphrey, 2012; Lanaj et al., 2016; Liang et al., 2016). Given that we explore general strain at work as a predictor (i.e., emotional exhaustion), we investigate negative affect (instead of specific negative emotions) as a mediator that captures the potentially broad affective response to emotional exhaustion.

**FIGURE 1:**
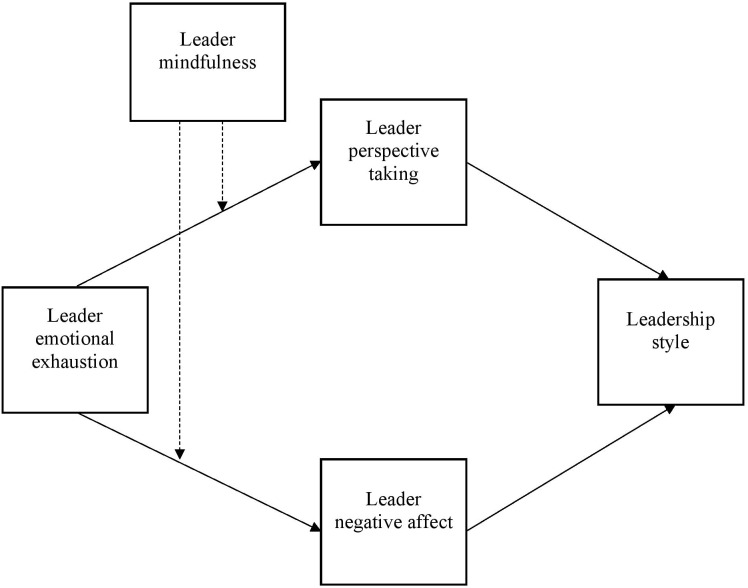
Visual summary of hypothesized model.

In terms of perspective taking, Glomb et al. (2011) suggest that the related construct of empathy is a resource that helps improve leadership behaviors specifically in relation to mindfulness. Although Glomb et al. (2011) note that empathy is important for organizational members at all levels, they, and others (e.g., Kellett et al., 2002; Scott et al., 2010) suggest that it its social functions make it particularly useful in improving leadership behaviors. Although empathy has both cognitive and affective components (e.g., Davis, 1980), we focus on perspective taking as a cognitive component of empathy to represent this important element of a leaders’ reflective system (Davis, 1980). We define perspective taking as “a cognitive attempt to consider another’s viewpoint” (Longmire and Harrison, 2018, p. 894). Recent research has suggested that perspective taking is indeed a distinct cognitive component of empathy (Longmire and Harrison, 2018), which aligns with dual process models of self-regulation and the importance of focusing on the needs of others in leadership positions. Please see Figure 1 for a visual representation of our hypotheses, which we will now discuss in more detail below.

### Negative Affect

Although previous research has not focused on emotional exhaustion as an antecedent to transformational leadership, we suggest that leaders who are emotionally exhausted would have a lower likelihood of transformational behaviors in comparison to leaders who are less emotionally exhausted (Byrne et al., 2014). Leaders who are emotionally exhausted would be ill-equipped to invest energy into the many positive behaviors transformational leadership requires. For example, transformational leadership requires positive affect (Bono et al., 2007) and emotional intelligence (defined as the ability to accurately perceive and control one’s emotions; Hur et al., 2011) which suggest that these positive behaviors require a strong reservoir of personal resources. Furthermore, some studies have found a positive relationship between emotional exhaustion and abusive supervision (Lam et al., 2017). Others have found a relationship between leader stress and abusive supervision (Zhang and Bednall, 2016), which suggest that both stress and strain outcomes (i.e., emotional exhaustion) are associated with abusive supervision.

The impulsive (i.e., affective) response to experiencing emotional exhaustion can explain *why* having high levels of emotional exhaustion would predict lower levels of transformational leadership and higher levels of abusive supervision. Experiencing emotional exhaustion can activate the impulsive system automatically. When experiencing emotional exhaustion, leaders can experience negative affect in a ‘bottom up’ fashion, where individuals experience emotional exhaustion and feel negative affect as part of a cyclical response to this exhaustion and its outcomes (Tugade, 2010). Thus, emotional exhaustion will predict higher levels of negative affect over time without conscious awareness. Research has supported the idea that negative affect is closely tied to ongoing strain, as experiencing an ongoing lack of need fulfillment can predict negative affect (Lanaj et al., 2016). Given that work-related emotional exhaustion similarly entails chronically negative thoughts about one’s job, it is likely that negative affect would be activated in an implicit way when experiencing emotional exhaustion.

In turn, negative affect is largely incompatible with the style of transformational leadership and is predictive of abusive behavior (Humphrey, 2012). Lanaj et al. (2016) found that transformational leadership is positively related to positive affect and is negatively related to negative affect. Other research has similarly confirmed that transformational leadership hinges primarily on positive affect (Carleton et al., 2018). Experiencing negative affect would be an automatic reaction to exhaustion that would be incompatible with a transformational leadership style and would explain why exhausted leaders are less likely to enact transformational behaviors. In contrast, a meta-analysis found negative affect predicts abusive supervision (Zhang and Bednall, 2016). Others have found that supervisor irritation (a negative emotion), predicts abusive supervision (Pundt and Schwarzbeck, 2018). Furthermore, feelings of hostility contribute to abusive supervision, and this type of negative emotion can be caused by sources of strain at work such as poorly performing subordinates (Liang et al., 2016). In turn, it is likely that the negative affect caused by emotional exhaustion could similarly predict abusive supervision (Zhang and Bednall, 2016). Overall, feeling emotionally exhausted increases negative affect, which is a key aspect of abusive supervision and is incompatible with transformational leadership.

However, mindfulness should moderate the relationship between leader emotional exhaustion and negative affect. As Glomb et al.’s (2011) framework suggests, mindfulness will disrupt the automatic tendency for emotional exhaustion to predict higher levels of negative affect for leaders. Being mindfully aware would reduce leaders’ automaticity, which would predict lower levels of negative affect for mindful leaders in comparison to less mindful leaders following the experience of emotional exhaustion.

Research further suggests mindfulness moderates the impulsive system. Mindfulness has been shown to buffer the relationship between aversive stimuli/chronic triggers of negative affect and intense affective reactions (Chiesa et al., 2013). Research has shown that mindfulness moderated the relationship between anxiety-related tasks and negative affect (Arch and Craske, 2010). In addition, mindfulness interacts with stressors such as injustice to predict lower levels of negative emotion (Long and Christian, 2015). In a similar vein, Glomb et al. (2011) found that the chronic strain of experiencing harsh environmental conditions at work was agitating for employees, but that practicing mindfulness allowed employees to regulate this ongoing agitation. These findings suggest that leader mindfulness similarly has the potential to interact with emotional exhaustion to ultimately predict less intense negative affect.

Research within neuroscience also supports the idea that mindfulness buffers automatic affective responses to emotional exhaustion. Taylor et al. (2011) compared the neural activity of novice and long-term mindfulness meditators while viewing emotional pictures. It was found that long-term meditators had reduced activity in the amygdala (an area of the brain that is known for generating affect) when viewing the pictures (Taylor et al., 2011). With reduced activity in this area while experiencing an emotional stressor, there is compelling evidence that mindfulness could similarly disrupt the impulsive system by moderating the relationship between ongoing emotional exhaustion and leaders’ automatic affective response. This suggests that with a strong present moment awareness, the relationship between acute stressors and impulsive (i.e., affective) reactions are buffered. Furthermore, mindfulness has also been shown to dampen negative emotion for leaders in longer term contexts (e.g., Liang et al., 2016), which suggests that more mindful leaders would also be likely to regulate their negative affect when experiencing the chronic strain of emotional exhaustion. Thus, we hypothesize the following:

Hypothesis 1a: The negative relationship between leader emotional exhaustion and transformational leadership is mediated by enhanced leader negative affect

Hypothesis 1b: The positive relationship between leader emotional exhaustion and abusive supervision is mediated by enhanced leader negative affect

Hypothesis 1c: The positive relationship between leader emotional exhaustion and negative affect is weaker for leaders who are high in mindfulness

In addition to the automatic processing of negative affect through the impulsive system, we suggest that the reflective system and the leaders’ perspective taking plays a potentially explanatory role in this relationship.

### Perspective Taking

Research on emotional exhaustion shows that it negatively predicts the use of cognitive resources such as perspective taking. For example, emotional exhaustion has been linked to cognitive failures, such as forgetting (van der Linden et al., 2005), in daily life for both working and non-working individuals. Interestingly, this study also showed that during tasks that require attentional focus, emotional exhaustion led to inhibition errors and decreases in performance (van der Linden et al., 2005). This suggests that in a leadership role, which requires focus and attention, emotional exhaustion would be likely to compromise cognitive resources such as perspective taking. Furthermore, in a review of studies of medical students and residents, the over-arching construct of empathy (of which perspective taking is a key component) was shown to decline over training periods because of the distress students experienced as part of the curriculum (Neumann et al., 2011). Emotional exhaustion, as an indication of strain emanating from chronic stress, would similarly impede a leader’s ability to take their followers’ perspectives because experiencing exhaustion would deplete cognitive resources making leaders less able to put themselves in someone else’s shoes. Overall, research suggests that experiencing emotional exhaustion would impair perspective taking as a cognitive resource.

Reduced perspective taking in response to emotional exhaustion can help subsequently explain why emotional exhaustion has a negative association with transformational leadership and a positive relationship with abusive supervision. Considering first the style of transformational leadership, there is evidence to suggest that the low levels of perspective taking experienced by emotionally exhausted leaders would predict a lower likelihood of enacting transformational leadership. Perspective taking was found to partially predict transformational leadership in previous research (Gregory et al., 2011). Theoretically, the transformational model is also based closely on developing high quality relationships with followers which links closely to taking the perspective of followers. The dimension of individual consideration, for example, requires a leader to recognize followers’ needs for development and support at work (Rafferty and Griffin, 2006). Given that perspective taking requires leaders to think about how others are responding to various situations at work, it is likely that a leader with high levels of perspective taking would more easily be able to enact individual consideration by putting themselves in their followers’ shoes. As another example, inspiring followers through a vision may also require perspective taking as a leader would be most inspirational when they are able to tailor the message of a vision to what is most important from the perspectives of followers (Stam et al., 2009). Furthermore, to enact intellectual stimulation, perspective taking may help a leader to see obstacles at work from their employees’ perspectives. By seeing problems from an employees’ point of view, a leader would be better able to recognize the resources and limitations an employee faces in solving that problem and would be better equipped to stimulate creativity in appropriate ways.

In contrast, the low levels of perspective taking that occur in the reflective system for emotionally exhausted leaders could explain why emotional exhaustion predicts abusive supervision. Although fewer studies focus on the antecedents of abusive supervision in comparison to its outcomes, one study has shown that perceived deep-level dissimilarity predicts abusive supervision (Tepper et al., 2011). This finding suggests that abusive supervisors psychologically distance themselves from followers, which is incompatible with perspective taking. Research also shows that the degree to which supervisors have a hostile attribution bias predicts higher abusive supervision when leaders experience a psychological contract breach (Hoobler and Brass, 2006). In other words, leaders who tend to attribute negative interactions to hostile motivations of others will be likely to be abusive. If leaders were high in perspective taking, in contrast, they would be less likely to automatically attribute negative interactions with followers as hostile (e.g., by putting themselves in their followers’ shoes) and would subsequently be less abusive. Overall, we propose that the relationship between leader emotional exhaustion and both transformational leadership and abusive supervision can be partly explained by the low levels of perspective taking that come from experiencing ongoing emotional exhaustion in a leadership role. Without the ability to invest cognitive resources into perspective taking, leaders experiencing ongoing emotional exhaustion are less able to identify with followers in a transformational way, and are more likely to lash out at followers who they are not identifying with.

However, mindfulness has the potential to weaken the relationship between leader emotional exhaustion and perspective taking. Glomb et al.’s (2011) framework suggests that in addition to inhibiting automatic mental processes (impulsive system), mindfulness also allows individuals to decouple themselves from immediate experiences, which means that mindful individuals are able to detach from self-oriented thoughts and experiences. In turn, this decoupling would predict less dramatic decreases in perspective taking for leaders following emotional exhaustion.

Empirical work also supports the proposition that mindfulness should moderate the relationship between emotional exhaustion and perspective taking. The negative relationship between the stress of an exam period and empathy was buffered for medical students who completed mindfulness training (Shapiro et al., 1998). Although perspective taking is one component of empathy, these findings suggest that mindfulness can similarly buffer the chronic strain of emotional exhaustion to promote less drastic decreases in perspective taking for emotionally exhausted leaders.

Second, mindfulness has been shown to increase meta-cognitive awareness (Glomb et al., 2011). Mindfulness-based practices often focus on gaining the ability to merely notice and accept one’s thoughts objectively, which creates meta-awareness of one’s thoughts and feelings (Glomb et al., 2011). With a mindful awareness, the relationship between leader emotional exhaustion and perspective taking would be weakened for mindful leaders, as mindfulness would promote the metacognitive awareness they need to imagine themselves in others’ situations despite the emotional exhaustion they feel. Instead of focusing on one’s self when emotionally exhausted, mindfulness would interact with emotional exhaustion to allow leaders to maintain a focus on others. Taken together, we hypothesize the following:

Hypothesis 2a: The negative relationship between leader emotional exhaustion and transformational leadership is mediated by reduced leader perspective taking

Hypothesis 2b: The positive relationship between leader emotional exhaustion and abusive supervision is mediated by reduced leader perspective taking

Hypothesis 2c: The negative relationship between leader emotional exhaustion and perspective taking is weaker for leaders who are high in mindfulness

A key aspect of Glomb et al. (2011) self-regulatory framework and general dual process theory is that both the impulsive and reflective systems operate in parallel to predict specific behaviors (Strack and Deutsch, 2004). Based on Glomb et al.’s (2011) framework, we suggest that a high level of trait mindfulness would simultaneously buffer the relationship between leader emotional exhaustion and both negative affect and perspective taking. Given that leader emotional exhaustion is likely to have automatic and reflective consequences that in turn influence leader behavior, we hypothesize a moderated mediation model where the buffering role of mindfulness not only predicts changes associated with the initial response to emotional exhaustion, but also serves to weaken emotional exhaustion’s predicted relations with transformational leadership and abusive supervision through this initial buffering effect. Please see Figure 1 for a visual representation of this moderated mediation model.

Hypothesis 3a: Mindfulness moderates the indirect effect of leader emotional exhaustion on transformational leadership through enhanced negative affect and reduced perspective taking, such that the indirect effects are weaker when mindfulness is higher

Hypothesis 3b: Mindfulness moderates the indirect effect of leader emotional exhaustion on abusive supervision through enhanced negative affect and reduced perspective taking, such that the indirect effects are weaker when mindfulness is higher

## Materials and Methods

To test the hypotheses outlined above, we conducted a survey (two waves, 3 weeks apart) of organizational leaders.

### Sample

We recruited leaders using TurkPrime^1^, which is an online recruitment service where participants are recruited for a fee based on demographic needs. We compensated participants $2.00 for each survey. At Time 1, 3,300 participants qualified for the study based on being employed full time in a supervisory position or higher where people report directly to him/her. Of 3,300 qualified participants, 750 participated at Time 1. Based on past research (Liang et al., 2016) and recommendations from Meade and Craig (2012), we excluded participants based on careless responding, which we assessed using an attention check question (“Please select strongly agree to this question”), and survey durations that were too fast (less than 40% of the median time; McGonagle et al., 2016). We excluded 107 participants based on these criteria, which resulted in 643 participants being invited at Time 2. At Time 2, 536 participants responded (response rate: 83%), and 31 were removed based on careless responding, resulting in a final leader sample of 505. These percentages of careless responders were found to be acceptable based on past research (Liang et al., 2016).

Leaders had a mean age of 37.30 years (range 19–69 years), a mean tenure in their current supervisory position of 6 years (range 1–38 years), and a mean of 9.64 years (range 1–40) of total supervisory experience. Sixty percent of the sample were male and were from a broad range of industries. The most popular categories of industry were: IT (11%), education (9%), sales/retail (8%), production/manufacturing (5%), and health care (6%).

Several *a priori* steps were taken to address common method bias in our design, as suggested by Podsakoff et al. (2003, 2012). We separated our predictors from our outcomes in time by 3 weeks, ensured participants that the survey was anonymous, and randomized the order of questions (Podsakoff et al., 2003). Furthermore, we used only measures that have been validated in previous research using both self and other reports (Podsakoff et al., 2003), as will be seen below.

### Measures

#### Emotional Exhaustion (Time 1)

We measured emotional exhaustion using five items from the Maslach Burnout Inventory (Maslach et al., 1996). We asked participants to rate on a scale from 1 (a few times a year) to 7 (every day) how often they experience the feeling or attitude described. Example items are “I feel emotionally drained from my work” and “I feel used up at the end of the workday.” Alpha was 0.94.

#### Mindfulness (Time 1)

We measured mindfulness using 15 items from the Mindful Attention Awareness Scale (Brown and Ryan, 2003). We asked participants to rate on a scale from 1 (almost always) to 6 (almost never), how frequently they have each experience. An example item is “I find it difficult to stay focused on what’s happening in the present.” Alpha was 0.94.

#### Negative Affect (Time 1)

We measured negative affect using 10 items from Watson et al. (1988)’s Positive and Negative Affect Scale (PANAS). The PANAS asks participants to rate from 1 (very slightly or not at all) to 5 (extremely) the extent to which they felt certain ways (e.g., distressed, guilty) in the last 3 weeks. Alpha was 0.92.

#### Perspective Taking (Time 1)

We measured perspective taking using seven items from Interpersonal Reactivity Index (Davis, 1980). Participants rated from 1 (does not describe me well) to 5 (describes me very well) their thoughts and feelings in a variety of situations. An example item is “I sometimes find it difficult to see things from the ‘other guys’ point of view.” Alpha was 0.86.

#### Transformational Leadership (Time 2)

Self-reported transformational leadership was measured using 20 items from the Multifactor Leadership Questionnaire (Bass and Avolio, 2000). Participants were asked to rate from 0 (not at all) to 4 (frequently, if not always) how frequently they exhibit the behavior in each statement in relation to their direct reports. Sample items are: “I talk optimistically about the future” and “I get others to look at problems from many different angles.” Alpha was 0.93.

#### Abusive Supervision (Time 2)

Self-reported abusive supervision was measured using 15 items from Tepper (2000). Leaders were asked to answer questions about how frequently they engage in various behaviors with their subordinate ranging from 1 (I don’t ever use this behavior with them) to 5 (I use this behavior very often with them). An example item is “Give them the silent treatment.” Alpha was 0.96.

Our choice for self-reported leadership behavior is based on the growing recognition that leaders are often aware of their entire range of behaviors, as direct reports (or others, such as peers) may not always see every behavior that a leader engages in. In relation to emotional exhaustion and the mediating processes linking it with leader behavior in particular, several recent related studies have relied on leader self-reported behavior, as leaders own understandings of their behavior may be most relevant when considering their affective and cognitive processes (Lanaj et al., 2016; Liang et al., 2016; Arnold et al., 2017). Thus, we measure leader behavior from leaders’ own perspectives to capture their overall leadership experience in relation to emotional exhaustion and its mediating processes.

#### Controls

For each analysis, we controlled for supervisory experience, given that this has been shown to be significantly associated with leadership style and leaders’ abilities to handle strain at work (Courtright et al., 2014).

## Results

Based on theory and past practice we conducted separate analyses for each leadership style (transformational leadership and abusive supervision). Studies investigating transformational leadership and abusive supervision as outcomes have treated them separately (e.g., Byrne et al., 2014). Dual process models also tend to investigate single behaviors as outcomes (e.g., Long and Christian, 2015), and leadership theory suggests that leaders are unlikely to enact both transformational leadership and abusive supervision (e.g., Hancock et al., 2018). Furthermore, a two factor CFA of the leadership style items suggested that these self-rated leadership behaviors are separate constructs (χ^2^ = 1810.02, df = 559, *p* < 0.001 CFI = 0.88; RMSEA = 0.07; SRMR = 0.06).

To ensure the construct validity of the measures used in our study, we conducted a confirmatory factor analysis (CFA) of the items using mplus (version 7.2) software. Model 1 included: emotional exhaustion, perspective taking, negative affect, mindfulness and transformational leadership. Model 2: emotional exhaustion, perspective taking, negative affect, mindfulness and abusive supervision. The fit indices suggest a reasonable fit for Model 1 (χ^2^ = 3729.82, df = 1529, *p* < 0.001 CFI = 0.87; RMSEA = 0.05; SRMR = 0.06) and Model 2 (χ^2^ = 3555.05, df = 1264, *p* < 0.001 CFI = 0.88; RMSEA = 0.06; SRMR = 0.06). Although the chi square test is statistically significant for both models, the convergence of the other fit indices suggest a reasonable fit to the data.

Furthermore, we conducted confirmatory factor analysis to demonstrate the discriminant validity of our predictor and mediating variables (all measured at Time 1). A three factor model of the items (including perspective taking, negative affect and emotional exhaustion) provided good fit to the data (χ^2^ = 942.92, df = 206, *p* < 0.001 CFI = 0.90; RMSEA = 0.08; SRMR = 0.06, whereas a one factor model provided a poor fit to the data (χ^2^ = 3903.44, df = 209, *p* < 0.001 CFI = 0.48; RMSEA = 0.19; SRMR = 0.17). In comparing the models [χ^2^_difference_ (3) = 2960.52, *p* < 0.01], our results suggest that the predictor and mediators are separable.

To test our hypotheses, we used Andrew Hayes’ PROCESS macro^2^ (version 2.16.2, 2016) for SPSS which uses OLS regression to test mediation, moderation, and moderated mediation (in addition to direct effects). Bootstrapping (5,000 iterations) was used to test indirect effects, conditional indirect effects, and to produce 95% bias corrected confidence intervals. For our mediation hypotheses (Hypotheses 1a-b and 2a-b) we conducted the analysis using Hayes (2013) PROCESS Model 4. For moderation (Hypotheses 1c and 2c) and moderated mediation (Hypotheses 3a and 3b) we conducted the analysis using Hayes (2013) PROCESS Model 7. Unstandardized coefficients are reported. Means, standard deviations and correlations between all variables in the study are outlined in Table 1.

**Table 1:** Means, standard deviations, and intercorrelations among variables.

Variable	*M*	*SD*	1	2	3	4	5	6
1. Supervisory experience (T1)	9.64	7.15						
2. Emotional exhaustion (T1)	3.65	1.69	-0.02					
3. Mindfulness (T1)	4.26	1.04	0.10^∗^	-0.37^∗∗^				
4. Negative affect (T1)	1.70	0.73	-0.01	0.48^∗∗^	-0.40^∗∗^			
5. Perspective taking (T1)	3.87	0.74	0.03	-0.01^∗^	0.24^∗∗^	-0.18^∗∗^		
6. Abusive supervision (T2)	1.41	0.64	-0.08	0.21^∗∗^	-0.23^∗∗^	0.37^∗∗^	-0.38^∗∗^	
7. TFL (T2)	3.82	0.61	0.14^∗∗^	-0.17^∗∗^	0.29^∗∗^	-0.22^∗∗^	0.44^∗∗^	-0.23^∗∗^


*^∗^*p* < 0.05; ^∗∗^*p* < 0.01; TFL, transformational leadership; T1, Time 1; T2, Time 2; supervisory experience is measured in years.*

### Transformational Leadership

As can be seen in Table 2, Hypothesis 1a is supported, as negative affect mediated the relationship between emotional exhaustion and transformational leadership (point estimate: -0.02, CI: -0.03 to -0.0009). Regarding the role of perspective taking, the indirect effect of emotional exhaustion through perspective taking is significant for the outcome of transformational leadership (point estimate = -0.01, CI: -0.02 to -0.0004), supporting Hypothesis 2a.

**Table 2:** Direct and indirect effects model coefficients for effects of emotional exhaustion on transformational leadership (TFL).

Consequent															

	Negative affect (T1; M)					Perspective taking (T1; M)					TFL (T2; Y)				
			
Antecedent	Coeff	*SE*	*p*	LLCI	ULCI	Coeff	*SE*	*p*	LLCI	ULCI	Coeff	*SE*	*p*	LLCI	ULCI
Negative affect (T1; M)	–	–	–	–	–	–	–	–	–	–	-0.09	0.04	0.02	-0.16	-0.01
Perspective taking (T1; M)	–	–	–	–	–	–	–	–	–	–	0.34	0.03	<0.001	0.27	0.40
Emotional exhaustion (T1; X)	0.21	0.02	<0.001	0.17	0.24	-0.04	0.02	<0.05	-0.08	-0.00	-0.03	0.02	0.09	-0.06	0.00
Supervisory experience (T1; C)	-0.00	0.00	0.90	-0.01	0.01	0.00	0.00	0.48	-0.01	0.01	0.01	0.00	<0.01	0.00	0.02
	*R*^2^ = 0.23					*R*^2^ = 0.01					*R*^2^ = 0.23				
	*F*(2,500) = 74.69, *p* < 0.001					*F*(2,500) = 2.49, *p* = 0.09					*F*(4,498) = 37.98, *p* < 0.001				


*X, independent variable; Y, outcome; M, mediator; C, covariate; LLCI, lower level of confidence interval; ULCI, upper limit of confidence interval; T1, Time 1; T2, Time 2; supervisory experience is measured in years.*

### Abusive Supervision

Hypothesis 1b is supported (see Table 3), as the indirect effect of emotional exhaustion on abusive supervision through negative affect (point estimate = 0.05, CI: 0.03 to 0.08) is significant. Hypothesis 2b is also supported, as the indirect effect of emotional exhaustion on abusive supervision through perspective taking is significant (point estimate = 0.01, CI: 0.0008 to 0.02).

**Table 3:** Direct and indirect effects model coefficients for effects of emotional exhaustion on abusive supervision.

Consequent															

	Negative affect (T1; M)					Perspective taking (T1; M)					Abusive supervision (T2; Y)				
			
Antecedent	Coeff	*SE*	*p*	LLCI	ULCI	Coeff	*SE*	*p*	LLCI	ULCI	Coeff	*SE*	*p*	LLCI	ULCI
Negative affect (T1; M)	–	–	–	–	–	–	–	–	–	–	0.25	0.04	<0.001	0.17	0.33
Perspective taking (T1; M)	–	–	–	–	–	–	–	–	–	–	-0.27	0.03	<0.001	-0.34	-0.21
Emotional exhaustion (T1; X)	0.21	0.02	<0.001	0.17	0.24	-0.04	0.02	<0.05	-0.08	-0.00	0.02	0.02	0.33	-0.02	0.05
Supervisory experience (T1; C)	-0.00	0.00	0.90	-0.01	0.01	0.00	0.00	0.48	-0.01	0.01	-0.01	0.00	0.09	-0.01	0.00
	*R*^2^ = 0.23					*R*^2^ = 0.01					*R*^2^ = 0.24				
	*F*(2,499) = 74.56, *p* < 0.001					*F*(2,499) = 2.52, *p* = 0.08					*F*(4,497) = 39.83, *p* < 0.001				


*X, independent variable; Y, outcome; M, mediator; C, covariate; LLCI, lower level of confidence interval; ULCI, upper limit of confidence interval; T1, Time 1; T2, Time 2; T3, Time 3; supervisory experience is measured in years.*

### Moderation

**FIGURE 2:**
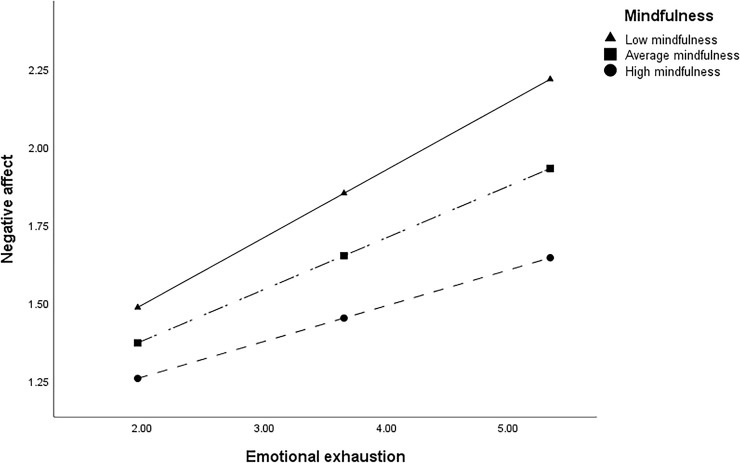
Moderating effect of mindfulness on the relationship between leader emotional exhaustion and negative affect.

Hypotheses 1c and 2c predict that the relationships between emotional exhaustion and (a) negative affect and (b) perspective taking are moderated by mindfulness, such that the relationships between emotional exhaustion and both negative affect and perspective taking are weaker for more mindful individuals. As shown in Table 4, the interaction between mindfulness and emotional exhaustion is significant for the outcome of negative affect (*b* = -0.05, *p* < 0.001) but not perspective taking (*b* = -0.02, ns). Thus, Hypothesis 1c is supported whereas Hypothesis 2c is not. A figure was produced to aid interpretation of the significant interaction (see Figure 2). As hypothesized, more mindful leaders reported experiencing lower negative affect despite their emotional exhaustion. Tests of simple slopes were conducted using Bonferroni adjusted alpha levels of 0.0166 per test (0.05/3), which reveals that this effect is indeed weaker for individuals high in mindfulness [*b* = 0.11, *t*(500) = 4.98, *p* < 0.001] than for those lower [*b* = 0.22, *t*(500) = 9.54, *p* < 0.001] or average [*b* = 0.17, *t*(500) = 9.57, *p* < 0.001] in mindfulness.

**Table 4:** Results for moderated mediation model.

Consequents						

	Negative affect (T1; M)			Perspective taking (T1; M)		
		
Antecedent	Coeff	*SE*	*p*	Coeff	*SE*	*p*
Emotional exhaustion (T1; X)	0.38	0.06	<0.001	0.10	0.08	0.18
Mindfulness (T1; W)	-0.01	0.06	0.83	0.25	0.07	<0.001
Mindfulness x Emotional exhaustion	-0.05	0.01	<0.001	-0.02	0.02	0.16
Supervisory experience (T1; C)	0.00	0.00	0.57	0.00	0.00	0.83
	*R*^2^ = 0.30			*R*^2^ = 0.06		
	*F*(4,498) = 54.62, *p* < 0.001			*F*(2,498) = 8.29, *p* < 0.001		

**Consequent: TFL (T2)**						

**Conditional indirect effects: Negative affect**						

**Variable**	**Indirect effect**			***SE***	**LLCI**	**ULCI**
Low mindfulness	-0.02			0.01	-0.03	-0.00
Average mindfulness	-0.01			0.01	-0.02	-0.00
High mindfulness	-0.01			0.00	-0.02	-0.00

**Conditional indirect effects: Perspective taking**						

Low mindfulness	0.00			0.01	-0.01	0.03
Average mindfulness	-0.00			0.01	-0.01	0.01
High mindfulness	-0.01			0.01	-0.02	0.00

**Consequent: Abusive supervision (T2)**						

**Conditional indirect effects: Negative affect**						

Low mindfulness	0.05			0.01	0.03	0.08
Average mindfulness	0.04			0.01	0.02	0.07
High mindfulness	0.03			0.01	0.01	0.06

**Conditional indirect effects: Perspective taking**						

Low mindfulness	-0.01			0.01	-0.02	0.01
Average mindfulness	0.00			0.01	-0.01	0.01
High mindfulness	0.01			0.01	-0.00	0.02


*X, independent; Y, outcome; M, mediator; C, covariate; LLCI, lower level of confidence interval; ULCI, upper limit of confidence interval; T1, Time 1; T2, Time 2.*

### Moderated Mediation

Finally, Hypothesis 3a predicted that mindfulness moderates the indirect effects of emotional exhaustion on transformational leadership negative affect and perspective taking. Hypothesis 3a is partially supported, as the conditional indirect effect between emotional exhaustion and transformational leadership through negative affect is weaker for leaders high in mindfulness (point estimate = -0.01, CI: -0.02 to -0.0023) than for those low in mindfulness (point estimate = -0.02, CI: -0.03 to -0.0019). However, given that Hypothesis 2c is not supported, the indirect effect through perspective taking is not moderated by mindfulness.

Hypothesis 3b was partially supported, as the conditional indirect effect between emotional exhaustion and abusive supervision through negative affect is weaker for those high in mindfulness (point estimate = 0.03, CI: 0.01 to 0.06) compared to those low in mindfulness (point estimate = 0.05, CI: 0.03 to 0.08).

## Discussion

The purpose of this study was to investigate whether leader mindfulness moderates the relationship between leader emotional exhaustion and its mediating processes (negative affect and perspective taking), to ultimately predict weakened relationships between emotional exhaustion and self-rated leadership behavior. Using a dual process framework of self-regulation, we tested whether leader perspective taking and negative affect mediated the relationships between emotional exhaustion and self-reported leadership behavior (transformational leadership and abusive supervision). We found support for our mediation hypotheses. Leaders emotional exhaustion predicted higher levels of abusive supervision and lower levels of transformational leadership, which is explained by the lower levels of perspective taking and higher levels of negative affect that were predicted by emotional exhaustion. In addition, mindfulness buffers the impulsive component of the dual process model (i.e., moderates the relationship between emotional exhaustion and negative affect), which also weakened the mediated link between emotional exhaustion and self-rated leadership style. The positive relationship between leader emotional exhaustion and negative affect was weaker for leaders who were higher on mindfulness compared to leaders lower on mindfulness. This initial interaction between mindfulness and emotional exhaustion, predicting lower levels of negative affect, served to weaken the negative relationship between emotional exhaustion and transformational leadership. Similarly, it weakened the positive relationship between emotional exhaustion and abusive supervision. In other words, emotional exhaustion still predicted self-rated abusive supervision and transformational leadership in expected directions, but these relationships were weaker for leaders who were high on mindfulness in comparison to leaders who were less mindful.

### Theoretical Implications

Theoretically, this study makes several key contributions. As outlined in our introduction, there has been a growing interest in understanding the relationship between mindfulness and leader behavior. Although research has shown direct relationships between leader mindfulness and leader behavior (e.g., Lange et al., 2018), we have demonstrated that mindfulness weakens the relationship between leader emotional exhaustion and leader behavior by buffering the relationship between emotional exhaustion and leaders’ negative affect. These findings advance understandings of mindfulness as having self-regulatory functions for leaders in particular, and complements existing research on leadership and mindfulness. Previous research on mindfulness has demonstrated that mindfulness moderates the relationship between stressors/strain and affect in many contexts (e.g., Long and Christian, 2015), and we extend and apply these findings to a leadership context. This has implications for further research on leadership and relevant self-regulatory processes. For instance, there is a growing recognition that many leader behaviors involve emotional labor, which can predict leader burnout (Arnold et al., 2015). Our work suggests that mindfulness could play a role in similarly reducing the negative effects of emotional labor by dampening the negative affect a leader feels to make ‘faking’ or suppressing emotions potentially easier in a complex leadership role.

In turn, this study contributes to the literature on mindfulness by demonstrating its importance for leaders’ affective regulation. This supports the growing recognition that the positive outcomes of mindfulness found in past research can potentially be explained by its self-regulatory capabilities (Glomb et al., 2011). A bulk of research on mindfulness in clinical psychology has demonstrated that mindfulness interacts with stress (and related strain outcomes such as depression), making affective reactions less intense through reduced automaticity (Chiesa et al., 2013). Instead of removing the stress and/or strain itself, Glomb et al. (2011) framework suggests that it is through these secondary regulatory processes that mindfulness is likely to have positive impacts in the workplace. Leadership roles are considered inherently stressful, and are likely to continue to be stressful as our economy grows and becomes more complex (Roche et al., 2014). Thus, the ability for mindfulness to predict dampened affective responses in leaders self-perceived behavior (as shown through our findings) is promising as the stress and strain of leadership (and in turn, emotional exhaustion) is likely to continue.

In terms of affect, these findings support previous studies that have found mindfulness moderates the relationship between negative workplace experiences and affect/emotion (Taylor et al., 2011; Long and Christian, 2015). Our findings indicate that more mindful leaders are able to maintain a higher level of transformational leadership and a lower level of abusive supervision than less mindful leaders, despite being emotionally exhausted. In this study, we investigated a range of negative affect as one construct, and a promising area for future research would be to investigate discrete emotions^3^ as mediators between various indications of leader resource depletion and leader behavior. Liang et al. (2016) found that leader mindfulness buffered the relationship between leader hostility and abusive supervision, and other studies of mindfulness at work have investigated whether mindfulness buffers the relationship between injustice and anger (Long and Christian, 2015). Future research should investigate these types of discrete emotions^3^, particularly anger as this is closely related to issues leaders must address such as poorly performing subordinates and lack of job control (Li et al., 2018). However, it would also be fruitful to take a positive approach and investigate whether mindfulness might also help to boost leaders’ positive emotions to improve leader behavior. Is it the case, for example, that mindfulness does more to dampen negative emotion than it does to build and maintain positive emotions such as joy?

In addition to demonstrating the self-regulatory functions of leader mindfulness, this is the first study to our knowledge to apply a dual process model specifically to mindfulness, emotional exhaustion and leadership. This model shows *why* leader resource depletion predicts negative leadership styles, and gives a new perspective on *how* this relationship can be positively disrupted. Specifically, the support we found for the dual process mediation model suggests that there are potentially both cognitive and emotional processes that can explain why a leader is likely to lash out at followers and be abusive when emotionally exhausted, in addition to why an emotionally exhausted leader is less able to invest in transformational leadership. By applying a dual process framework, we show that the relationship between leader emotional exhaustion and leadership style likely hinges on leaders’ cognitive and affectual mediating processes.

Finally, this study contributes to the growing literature on leadership and emotional exhaustion. Recent stress and strain research has shifted focus to the leader’s perspective (Harms et al., 2017; Li et al., 2018), as there has been a growing recognition that leaders’ experiences at work are distinct from employees’ experiences. This study further confirms the negative association between leader emotional exhaustion and positive leadership styles suggesting that leaders who are exhausted are less able to invest in transformational leadership and are more likely to abuse their followers. Overall, these findings suggest that a leaders’ emotional exhaustion can make the difference between a work environment where leaders perceive themselves as motivating and empowering, versus one where leaders perceive themselves as abusive toward followers.

However, our results suggest that mindfulness only moderates the relationship between emotional exhaustion and negative affect (the impulsive system) and not perspective taking (the reflective system). There are two key theoretical reasons why this may be the case, both of which raise important questions for future research. In comparison to the impacts of mindfulness on affective processing, relatively few studies have investigated the benefits of mindfulness on aspects of the reflective system that improve interpersonal relationships, such as perspective taking. Perspective taking is a positive cognitive process that is related to understanding others, whereas applications of dual process models related to mindfulness in business research have often examined negatively focused elements of the reflective system such as rumination and hostility (e.g., Long and Christian, 2015). It could be the case that mindfulness does more to help build buffer the relationship between strain and negative states of mind by detaching, than it does to amplify the positive elements of these systems. Future research is necessary to further compare and contrast perspective taking with other elements of the reflective system to determine how and why mindfulness might regulate this type of cognition.

Furthermore, it could be the case that the level of leadership that is being investigated may play a role in whether the relationship between stress/strain and perspective taking is buffered by leaders’ mindful awareness. We investigated relatively early career leaders who were in a direct supervisory position or higher; it could be the case that in higher leadership positions perspective taking may play a more prominent role in activities such as strategic decision making with followers best interests in mind. It would be helpful for future studies to look at leadership in specific levels and contexts to better understand how mindfulness may play a more important role when understanding and recognizing the needs of others is a prominent focus of both the job and ones’ leadership role.

### Practical Implications

This study also brings forward practical implications for addressing leader emotional exhaustion at work. First, the findings from this study further support the importance of leaders’ well-being for leaders’ self-rated behavior. Although leaders are often encouraged to support employee well-being, this study shows that when emotionally exhausted, leaders may be poorly equipped to do so. It is thus important for leaders on an individual level to be aware of how their own well-being may be impacting their behaviors at work, and to seek resources on a personal or organizational level to maintain leadership effectiveness.

Second, organizations should be aware of the detrimental impact leader emotional exhaustion can have on leadership style and aid leaders in building personal resources. In particular, our findings suggest that mindfulness may be a resource that helps leaders maintain leadership effectiveness even when experiencing emotional exhaustion. Mindfulness training programs have been shown to be an effective intervention for improving mindful awareness in many contexts (Brown and Ryan, 2003), so this is one strategy organizations could use to address the potentially negative consequences of leader emotional exhaustion. There are also a broad range of mindfulness programs to consider (e.g., MBSR, short smartphone-based guided meditation), so leaders can realistically cultivate their mindfulness in flexible ways that best fit their busy lifestyles and needs.

### Limitations

This study has some limitations that should be acknowledged. First, we used self-report data from leaders as our dependent variable. Although some research suggests that leaders may over-report relationship-oriented behaviors (Lee and Carpenter, 2018), we had intented to capture leadership from leaders’ own perspectives (see “Materials and Methods” section). Leaders’ self-reported leadership style has been used in other studies focusing on leader affect and well-being (Lanaj et al., 2016; Liang et al., 2016; Arnold et al., 2017), which would suggest that when it comes to leader emotional exhaustion and well-being, how a leader perceives him/herself may be particularly relevant. It is also worth noting that self and other ratings are similar when considering peer and superiors’ ratings and the purpose of the ratings (Lee and Carpenter, 2018). Thus, it is plausible that leaders are in a position to elicit reliable judgments of their own behavior, as followers may not see some of the behaviors a leader is aware of. Nonetheless, a majority of leadership research does use follower reports of leader behavior, so future research should investigate whether our findings regarding the self-regulatory role of leader mindfulness would be similar when leader behavior is measured from the perspective of followers.

Second, this is a cross-sectional study with two measurement points. Thus, we are unable to test mediation using three different time points and it is possible that common method bias has influenced the results. In terms of common method bias, as noted in the previous section, we have followed suggestions from Podsakoff et al. (2012) to address common method bias: We collected data on the predictors and criteria at separate times, used an anonymous survey, randomly ordered questions, and used measures that have been validated in previous research. In terms of mediation, we have not controlled for baseline levels of both our mediators and outcomes given that we have data at only two time points (Cole and Maxwell, 2003). We do believe, however, that these results demonstrate potentially important relationships between leader mindfulness and emotional exhaustion/leadership style that can be explored further in future studies.

Third, the non-experimental nature of the study design does not allow for causal inferences to be made. Many studies of dual process self-regulation models have been experimental (e.g., Long and Christian, 2015), so future research using an experimental approach would address this limitation. Although it is not possible to manipulate emotional exhaustion, an experimental approach could be applied when examining related constructs that might predict emotional exhaustion when repeated over time, such as task stress within a leadership context.

Taking these limitations into account, the positive findings regarding mindfulness from the current study could inspire future replication and extension studies using a variety of methodological approaches. One promising avenue would be to use intensive longitudinal designs (e.g., diary study) of leaders to allow for a rigorous analysis of how within-leader fluctuations in emotional exhaustion (or related constructs such as stress) interact with daily fluctuations in mindfulness^4^. Self-regulation is ongoing throughout the workday, and other studies of leader self-regulation have demonstrated the rich insights that can be gained from this approach (e.g., Barnes et al., 2015). Overall, this would allow for a better understanding of the psychological processes that explain how and why leader mindfulness improves leader behavior.

## Conclusion

Overall, this study sought to examine the relationship between emotional exhaustion and leadership style using a dual process framework of self-regulation. Using leader self-reports, we found that negative affect and perspective taking mediated the relationship between leader emotional exhaustion and leadership style (transformational leadership and abusive supervision), and that mindfulness buffers the relationship between emotional exhaustion and negative affect. The moderation of the relationship between emotional exhaustion and negative affect ultimately predict weakened mediated relationships between emotional exhaustion and leadership style. This study demonstrated the potential importance of mindfulness in helping leaders maintain higher levels of transformational leadership and lower levels of abusive supervision despite feeling emotionally exhausted in their demanding roles.

## Ethics Statement

This study was carried out in accordance with the recommendations of the Tri-Council Policy Statement on Ethical Conduct for Research Involving Humans 2 (TCPS2). The protocol was approved by the Interdisciplinary Committee on Ethics in Human Research (ICEHR). All subjects gave written informed consent in accordance with TCPS2.

## Author Contributions

MW collected and analyzed the data. KA assisted with theoretical development and data analysis.

## Conflict of Interest Statement

The authors declare that the research was conducted in the absence of any commercial or financial relationships that could be construed as a potential conflict of interest.

## Footnotes

1 www.turkprime.com

2 www.afhayes.com

3 We would like to thank the reviewer for this suggestion.

4 We would like to thank the reviewer for this suggestion.
